# Adapting a large database of point of care summarized guidelines: a process description

**DOI:** 10.1111/jep.12426

**Published:** 2015-08-07

**Authors:** Nicolas Delvaux, Stijn Van de Velde, Bert Aertgeerts, Martine Goossens, Benjamin Fauquert, Ilka Kunnamo, Paul Van Royen

**Affiliations:** ^1^ Academic Centre for General PracticeDepartment of Public Health and Primary CareKatholieke Universiteit LeuvenLeuvenBelgium; ^2^ EBM*Practice*NetLeuvenBelgium; ^3^ Belgian Centre for Evidence Based Medicine (CEBAM)LeuvenBelgium; ^4^ DuodecimHelsinkiFinland; ^5^ Department of Primary and Interdisciplinary Care AntwerpUniversity of AntwerpAntwerpBelgium

**Keywords:** evidence‐based medicine, knowledge bases, point‐of‐care systems, practice guidelines as topic

## Abstract

**Rationale, aims and objectives:**

Questions posed at the point of care (POC) can be answered using POC summarized guidelines. To implement a national POC information resource, we subscribed to a large database of POC summarized guidelines to complement locally available guidelines. Our challenge was in developing a sustainable strategy for adapting almost 1000 summarized guidelines. The aim of this paper was to describe our process for adapting a database of POC summarized guidelines.

**Methods:**

An adaptation process based on the ADAPTE framework was tailored to be used by a heterogeneous group of participants. Guidelines were assessed on content and on applicability to the Belgian context. To improve efficiency, we chose to first aim our efforts towards those guidelines most important to primary care doctors.

**Results:**

Over a period of 3 years, we screened about 80% of 1000 international summarized guidelines. For those guidelines identified as most important for primary care doctors, we noted that in about half of the cases, remarks were made concerning content. On the other hand, at least two‐thirds of all screened guidelines required no changes when evaluating their local usability.

**Conclusions:**

Adapting a large body of POC summarized guidelines using a formal adaptation process is possible, even when faced with limited resources. This can be done by creating an efficient and collaborative effort and ensuring user‐friendly procedures. Our experiences show that even though in most cases guidelines can be adopted without adaptations, careful review of guidelines developed in a different context remains necessary. Streamlining international efforts in adapting international POC information resources and adopting similar adaptation processes may lessen duplication efforts and prove more cost‐effective.

## Introduction

A key step in implementing evidence‐based medicine (EBM) is providing the right information to the right care provider for the right patient [1, 2, 3]. For this information, doctors need access to information resources capable of answering health questions posed at the point of care (POC). Efforts to define doctors' information needs show that POC information resources should be comprehensive, electronic and able to answer complex questions [4, 5, 6]. Promising examples are the resources defined by Banzi *et al*. [7] as web‐based medical compendia specifically designed to deliver predigested, rapidly accessible, comprehensive, periodically updated and evidence‐based information and guidance to clinicians [8, 9].

As a part of a broader national eHealth strategy, the Centre for Evidence Based Medicine (CEBAM) and other Belgian EBM organizations founded a national electronic POC information service named EBM*Practice*Net in 2011. Its aim was to promote evidence‐based decision making and ultimately to optimize quality of care [10]. It is funded by the Belgian national health insurance institute (National Institute for Health and Disability Insurance, INAMI‐RIZIV). An inventory of information resources available to Belgian doctors shows they can rely on about 50 Belgian clinical practice guidelines (CPGs) of good quality linked to electronic health records (EHRs) through the Evidence Linker [11], a tool that automatically suggests guidelines and evidence‐based information based on the patient's diagnoses. One of the disadvantages of using CPGs at the POC is their length and layout. In contrast, through their summarized format and user‐friendly interface, POC information resources have a place higher up the pyramid designed to aid information retrieval for complex questions as defined in the 6S model by Haynes [12, 13, 14]. Developing a new database of POC summaries was impossible because of limited resources. EBM*Practice*Net therefore chose to subscribe to a POC information resource, supplementing it with existing Belgian guidelines in a reformatted and summarized format, and making it accessible to all Belgian health care professionals. In a first stage, efforts were targeted at primary care doctors. Comparison between existing POC information resources showed that information quality can differ significantly [15]. EBM*Practice*Net subscribed to the Duodecim EBM Guidelines (EBMG) database [7, 16], containing almost 1000 international summarized guidelines, updated on a yearly basis with a full revision every 3 years. The choice for the Duodecim database was based on an appraisal of the evidence‐based methodology, size of the database, indexation characteristics, focus on primary care, editorial quality, updating speed, possibility to adapt the guidelines to the Belgian context and link with a decision support system [7, 10, 17]. Also important in this decision was the accreditation granted by the National Institute for Health and Care Excellence (NICE) assessing the methodology used by the authors of the guideline database [7].

Our challenge was to develop a process to locally adapt almost 1000 summarized guidelines as well as their yearly updates within a 4‐year period. By adaptation, we understand a systematic approach to consider the use or modification of (a) guideline(s) produced in one cultural and organizational setting for application in a different context [18]. Adapting foreign CPGs is an alternative to developing local CPGs while avoiding unnecessary efforts yet ensuring sufficient rigor of development [19, 20, 21, 22, 23]. As opposed to CPGs, for which there are formal adaptation processes, to our knowledge, there have been no previous efforts to adapt a database of summarized guidelines [18, 24]. We tailored an existing CPG adaptation method to evaluate the large set of international summarized guidelines and adapted them to a local context and involving a large number of collaborators. The aim of this paper was to describe a collaborative approach to adapt a large database of summarized guidelines and to evaluate its usability.

## Methods

### Resources

The founding members of EBM*Practice*Net, including CEBAM, are all organizations active in the production or dissemination of EBM information. The collaboration between EBMPracticeNet and Duodecim has previously been reported by Van de Velde *et al*. [10]. EBMPracticeNet is run by an editorial team of 1.8 full‐time equivalents (FTEs), consisting almost entirely in general practitioners (GPs). To increase capacity, we decided to involve a large number of participants in the adaptation of the EBMG database. First, we invited health care professionals with a background in developing or evaluating EBM information to cooperate. These participants included GPs, nurses, physiotherapists and occupational therapists with a background in developing EBM information such as CPGs or evidence summaries. Additionally, in collaboration with the medical faculties of various universities in Belgium, we invited GP trainees to dedicate their master thesis to the adaptation of an EBMG guideline. Finally, remaining guidelines were processed by the EBM*Practice*Net editorial team.

### Prioritization

In order to manage the large number of guidelines to be adapted, we first divided the EBMG database into low‐ and high‐priority guidelines based on their anticipated relevance for Belgian doctors. We considered guidelines to be of high priority if they were more relevant to doctors, meaning that they were more likely to be accessed or that using the guideline was anticipated to have a higher impact on health care. All other guidelines were considered as low‐priority guidelines. To determine the relevance of each guideline for the users, we analysed three data sources: (1) the EBMG user statistics for Finland; (2) the results of a Belgian study by Van Duppen *et al*. registering health questions of primary care doctors posed at the point of care [25]; and (3) the Belgian guidelines most frequently viewed by primary care doctors using the Evidence Linker within their EHR [11]. For each source, the data were translated to International Classification of Primary Care (ICPC‐2) codes [26] and compared. We used the results of these three sources to generate a list of high‐priority guidelines. Before commencing with the adaptation process, this list was presented to the EBM*Practice*Net editorial board for validation.

### Content evaluation

**Figure 1:**
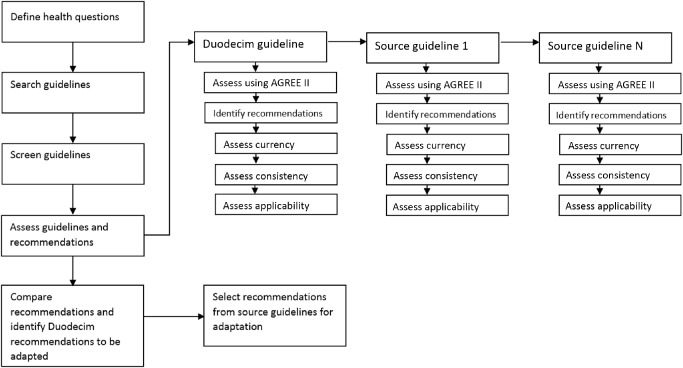
Schematic view of the adaptation process (based on the ADAPTE framework).

Based on the ADAPTE framework, we designed an adaptation handbook specifically for our project (Fig. 1). The ADAPTE framework has previously been presented in other publications [18, 27, 28]. It is regarded as a useful tool in assisting guideline adaptation and has been used to compare local processes as gold standard [23, 29].

Notwithstanding an almost identical approach to guideline adaptation, there were some clear differences. Whereas the ADAPTE framework starts with health questions and subsequently screens existing CPGs, our approach was in reverse order. We started from an existing EBMG guideline and first identified health questions from this document. Next, other CPGs were screened and recommendations to the identified health questions inventoried. Although the following steps of our local handbook were the same as those of the ADAPTE framework, the choice to adapt certain recommendations was different. After comparing the original EBMG guideline with other CPGs, we suggested adapting a recommendation only in case of incongruence. For each EBMG guideline, we recorded every step of the adaptation process in an Excel worksheet designed for this purpose, further referred to as a matrix (see Supporting Information Appendix S4 for an example). Matrices and remarks about the content were reported to Duodecim to be taken into consideration during their next updating cycle. For each screened Duodecim guideline for which a content adaptation was proposed, we wrote an editor's note that was added as a link to the online version of the guideline on EBM*Practice*Net. The proposed content remarks were screened by a member of the editorial board and a distinction was made between important notes, with possibly significant impact on the clinical situation, and less important notes. Given the rigor of this process and the amount of time needed to evaluate each guideline, this content evaluation was reserved for the high‐priority guidelines.

### Contextual evaluation

Alongside the content evaluation, we developed a process to evaluate the local applicability of the recommendations for each EBMG guideline. We referred to this step as contextual evaluation. All Duodecim guidelines underwent contextual evaluation, regardless of priority. The criteria used to assess the context included a review of recommended drugs and their local availability, differences in legislation that may interfere with the recommendations, differences in guidelines for referral, differences in resources and organization (such as the availability of nurses and other paramedics, day clinics), differences in transport modalities (such as distances to hospitals, centralization of advanced care) and differences compared with existing national guidelines or other evidence‐based practice. All comments were pooled in a specifically designed Excel sheet and important comments were added to the online text in the form of an editor's note.

### Quality assessment

For each completed matrix, the key remarks and suggestions for adaptations were reviewed by a member of the editorial board with experience in guideline development. The relevant key remarks were inventoried and translated into editor's notes and proofread before publication on the website. Supporting Information Appendix S2 shows an example of an editor's note. The GP trainees were coached and assessed more thoroughly than the more experienced participants. They received individual feedback throughout the adaptation process and their results were revised on two separate occasions by members of the editorial board before being published on the website as an editor's note.

## Results

### Prioritization

**Figure 2:**
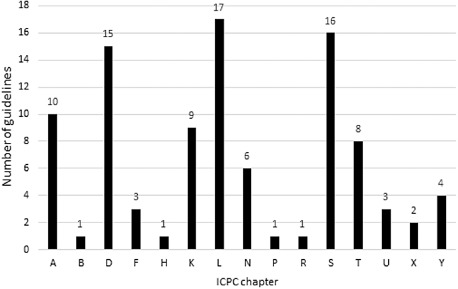
Distribution of high‐priority guidelines according to ICPC‐2 chapters.

Forty‐five EBMG guidelines were withdrawn from the database because Belgian guidelines were available for the same subject. Comparison of the EBMG user statistics with the Belgian data by Van Duppen *et al*. and the Evidence Linker user statistics showed a large similarity in the clinical topics accessed at the POC. Based on this finding, we selected the 97 most frequently consulted EBMG guidelines as the high‐priority collection to be adapted first and most thoroughly. Figure 2 shows the distribution of the high‐priority guidelines according to their ICPC‐2 chapter and Supporting Information Appendix S1 shows the titles of all high‐priority guidelines.

To validate our prioritization strategy, we cross‐checked our user statistics for 2014 with the list of high‐priority guidelines. Of the 100 most accessed guidelines, corrected for guidelines not prioritized because a Belgian version exists, 58 had been selected as high‐priority guidelines. The remaining 42 had been marked as low‐priority guidelines.

### Content and context evaluation

**Figure 3:**
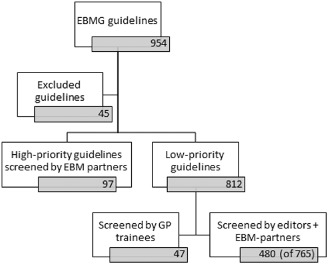
The amount of guidelines screened on content (evidence‐based content) and context (local applicability) from 2011 to 2014.

**Figure 4:**
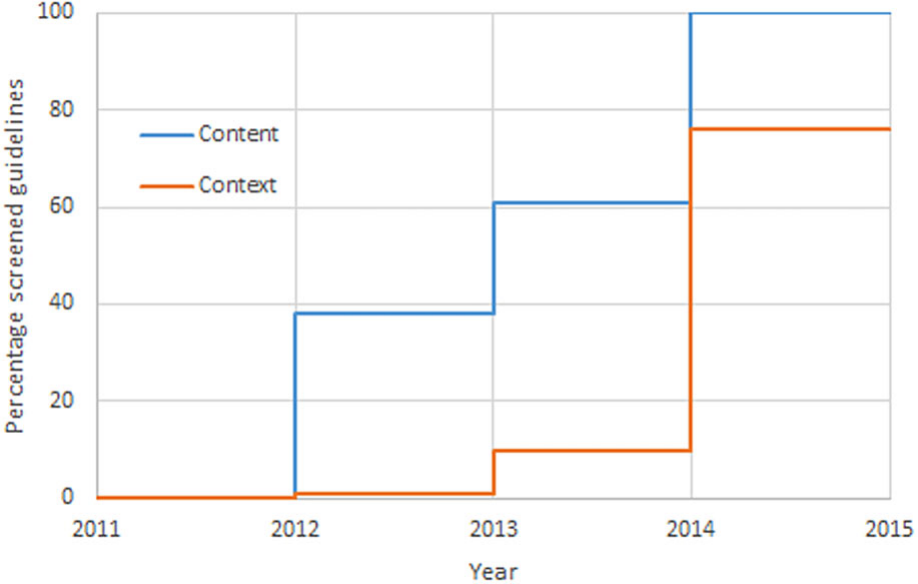
Results of adaptation efforts from 2011 to 2014.

The results for both the content and contextual screenings can be found in Table 1, Figure 3 and Figure 4). Between the end of 2011 and the end of 2014, the content of 97 high‐priority guidelines was assessed. This work was undertaken by 30 different participants from 15 different organizations. The mean time spent per guideline was 12.5 hours (ranging from 4 hours to 35 hours). Participants experienced in the use of the ADAPTE framework required less time for the adaptation process, with a mean time of 9.5 hours. 70% of the high‐priority guidelines required content adaptation. However, only in 9% of the guidelines was an important content remark, with possibly significant impact on the clinical situation, formulated. An important reason for clarifying recommendations was the difference in diagnostic and therapeutic responsibility for GPs in Belgium as compared with Finland. Although this could be considered a contextual difference, often there were evidence‐based reasons for quicker referral or performing diagnostic tests in secondary care. Another reason for adding an editor's note was because of the summarized format of the guidelines which sometimes did not allow for the necessary nuance. The summarized format favoured more radical recommendations even though the evidence base did not allow for it. Supporting Information Appendix S2 shows an example of a content related editor's note. We were unable to adapt a guideline on vaccinations. Local vaccination schemes differed significantly with those in the original guideline and it was therefore replaced by a local guideline. Over the last 3 years, 83 GP trainees had started the adaptation process for an additional 41 low‐priority guidelines. They assessed each guideline on clinical content and subsequently on local applicability.

**Table 1:** Results for the adaptation process for content (evidence‐based content) and contextual (local applicability) screening from 2011 to 2014

	Content adaptations		Context adaptations	
	Number of guidelines	Percentage (%)	Number of guidelines	Percentage (%)
Required no adaptations	29	30	533	66
Required adaptations	68	70	91	11
Minor adaptations	59	61		
Major adaptations	9	9		
Unscreened	0	0	188	23
Total	97		812	

Between the end of 2011 and the end of 2014, the local applicability of 577 guidelines was evaluated. Ninety‐one (11%) guidelines required contextual adaptations. The screening is still ongoing for 188 guidelines, but thus far, 533 guidelines have been identified that do not require any adaptation. The mean screening time per guideline was approximately 2 hours. It should be noted that the reviewers primarily screened those low‐priority guidelines where no conflicts of applicability were expected. The remaining guidelines to be reviewed should prove more complicated.

Frequent examples (see also Supporting Information Appendix S3) of contextual adaptation included changes in the recommendations on:

- Drugs: changes to drug recommendations were needed because certain drugs were not locally available or not available in the recommended dose. In case of recommendations on antibiotic prescription, adaptations were often made to better match local recommendations on antibiotic dosage [30].
- Diagnostic procedures: because of differences in health care organization, some diagnostic procedures recommended primary care, are locally performed in specialized care.
- Therapeutic procedures: because of the proximity of specialized care in Belgium some therapeutic procedures recommended in primary care, are locally assigned to specialized care.

## Discussion

Over a period of 3 years, we managed to screen and adapt about three‐quarters of the 1000 EBMG guidelines using an adaptation method based on the ADAPTE Manual. The screening process is still ongoing for one‐third of the guidelines that we considered low priority. Even though our screening process is still ongoing, our current results already provide considerable insight into the difficulties encountered on adapting a POC database of summarized guidelines. First, the remaining unscreened guidelines are all rarely accessed by GPs and will probably not add new insights. Furthermore, our process will remain an ongoing process because we will need to continue to screen and adapt future updates. In this sense, this process will never really be finished and results will, in a sense, remain preliminary ones.

### Limitations

A limitation of our method is that, because of limited resources, we have not yet been able to keep up with the EBMG updating cycles. First, the translations into Dutch and French have created a delay so that by the time a participant was requested to evaluate the content or context of the translated version, there was often a newer (English) version available. To avoid duplication, the participant was asked to evaluate, if available, the latest version of the EBMG guideline. The delay between receiving an updated guideline and its content or context assessment is an issue that warrants careful consideration. However, despite these obstacles, we foresee catching up with the EBMG updating by the end of 2015. In a recent review, of the five most comprehensive databases of POC information, EBMG was the fastest after Dynamed® to incorporate new evidence in their updates, but still it took about 10 months before a new systematic review is incorporated into their guidelines [17]. Translating and adapting these guidelines only adds to this delay and efficiency should be enhanced where possible.

### Improving the adaptation process

Our adaptation process has shown that the questions posed at the POC by Finnish GPs and Belgian GPs are similar but not identical. The cross reference of our user statistics for 2014 with the list of high‐priority guidelines, based on the Finnish user statistics in 2011, showed that for the 100 most accessed guidelines, a little more than half were considered high priority. Of the first 50 guidelines, almost all were high priority. Many of the highly accessed low‐priority guidelines were selected by GP trainees for their master thesis. The frequent consultation of the online guideline by the GP trainees is a plausible explanation for why these low‐priority guidelines were so frequently accessed. When determining which guidelines to adapt first in a large database of topics, at first, the user statistics of other countries are a valid instrument for cataloguing guidelines by priority. However, information needs between countries are not identical and local user statistics should be used to improve prioritization.

A survey by the Guideline International Network reported that the need for training, the complexity of the process and the time needed to adapt a CPG are perceived as barriers to the use of a formal adaptation process [31]. Very similar barriers were identified by Harstall *et al*. in the evaluation of the Alberta Ambassador Project [24, 29], by Chakraborty *et al*. [19] in Australia. They were very much the same as those expressed by the participants involved in our process. Most participants found the process clear but cumbersome. However, by providing a clear outlined process description in a handbook, we ensured that the quality of the end results of most participants was very similar despite the differences in their experience. This finding supports the experiences in Estonia that when using formal guideline development methods, small organizations, with limited resources, can develop high‐quality guidelines [32, 33].

One of the most frequent initial remarks made by the participants was the lack of formal training in the use of the handbook and the matrix. Many participants, though experienced in guideline development, had no previous experience of a formal adaptation process. Our results also show that there is a learning curve: participants experienced in the use of the ADAPTE Manual performed the task significantly quicker than inexperienced ones. In response to this observation, training sessions were offered. GP trainees involved in our process considered these training sessions to be very useful.

To increase the efficiency of the context adaptation, an instrument is being developed that creates a semi‐automatic link between the drugs mentioned in the Duodecim EBM Guidelines and the Belgian website for pharmacotherapeutical information (http://www.bcfi.be). This website offers information on available doses, packages, dosages, reimbursement requirements, indications and contraindications for all prescription and some over‐the‐counter drugs available in Belgium and links can be made with the EBM*Practice*Net guidelines. When they have been integrated in the published guidelines, these hyperlinks will also become available to the end‐users.

### Adaptation and implementation

Whether adapting guidelines is useful is still subject to discussion. There is little evidence that adapting guidelines improves their implementation. Eisenberg launched the idea that evidence may be global, but recommendations are local and suggests that guidelines are most successful when local differences have been taken into account in formulating recommendations [34]. Guyatt states that the strength of recommendations may differ according to the views and preferences of different patients in different settings, despite being based on the same strong evidence [35]. For Europe, it is impossible to develop recommendations that take into account the very diverse cultural, economic and social settings of all these countries. This presumption is also the basis of the ADAPTE framework. However, even though adapting high‐quality CPGs may not improve their implementation in daily practice, this is not necessarily so for POC summarized guidelines. By analysing the references of different POC information products, Ketchum illustrated that these products can vary greatly with regard to the amount of citations, the type of evidence and the currency of the cited evidence [15]. Adapting these summarized guidelines using a formal process that reviews the supporting evidence avoids implementing recommendations that are locally unacceptable. This context convinced us not to simply adopt the EBMG database but to invest resources into adapting them to the Belgian setting. Our efforts, however, have shown that after careful review, in a set of 750 international guidelines, only about a third of the guidelines will require either content or contextual adaptation. This may contradict Eisenberg's claim that evidence is global and recommendations local. Our experiences suggest that in many cases, not only the evidence, but also the recommendations, prove to be global and require no adaptation.

Since the start of our project, we have monitored the user statistics of our website and consider it an indicator of satisfaction with our project. Preliminary results (not reported here) show a steady increase in the amount of individual users accessing our website with 1405 unique users and 246 new users per month at the start of 2015. At present, 12% of the active Belgian GPs access our resource every month, but attributing this success entirely to our adaptation process would be unjust since we used a multifaceted implementation and dissemination strategy for our project. We plan to report on these strategies in the future.

### International collaboration

We conferred with Österreichische Gesellschaft für Allgemein‐ und Familienmedizin (ÖGAM), an Austrian medical society with several years of experience in the translation and adaptation of the EBMG guidelines [36] and with a Scottish branch of the National Health Service (NHS) that is piloting the clinical decision support tool EBMeDS [37] based on the EBMG guidelines. Their experiences have been a source of inspiration for our process. Streamlining the processes and sharing comments on guidelines may increase the efficiency of this effort and encourage other foreign organizations to do the same. We believe that the opportunity here lies in developing a database of high‐quality international guidelines, supported by a large body of international participants with a toolkit on how to adapt these guidelines for local use where necessary.

## Conclusions

Our efforts have shown that it is possible to use a formal adaptation process for the adaptation of a large POC database of summarized guidelines. We managed this with limited resources and without compromising on rigor of development. With a staff of almost two FTEs and many external participants we managed to critically appraise and adapt almost 750 out of 1000 summarized guidelines over a 3‐year period. Estonia, also a small country with limited resources, has already demonstrated that it is possible to develop high‐quality guidelines when using a formal adaptation process [33]. Our experiences strengthen this conclusion. In comparison, the National Clinical Guideline Centre of NICE employs a staff of almost 70 people (66.7 FTE) to develop and maintain almost 180 guidelines (source http://www.ncgc.ac.uk). However, the quality and comprehensiveness of the NICE guidelines is of a very different order. Adapting guidelines, however, implies that there are organizations such as NICE that provide in high‐quality guidelines that may be adapted by others. We would also like to emphasize that developing and maintaining a POC database of summarized guidelines remains a task with a significant impact on resources. It also requires experience in guideline development and a systematic approach to guideline evaluation and adaptation. It is therefore important that every organization planning to adapt foreign POC databases is experienced in guideline development and this expertise is maintained. Currently, more organizations are adapting POC databases [38, 39]. It may therefore be useful to develop strategies to stimulate participants and organizations in different countries to adopt a similar adaptation process. This may lessen duplication efforts and be more cost‐effective. We have invested a lot of resources into adapting these summarized guidelines. Further research will need to demonstrate the effect of these efforts on implementation. To ensure funding, the impact of an (adapted) POC CPG database on the quality of care will also need to be demonstrated. It would be of interest to determine the effect of these databases on patient care outcomes.

## Conflict of interests

SVDV is the salaried Project Leader and Editor‐in‐Chief of EBM*Practice*Net. BF is chairman and BA and ND are members of the EBM*Practice*Net board of directors. BF and MG are salaried editors for EBM*Practice*Net. ND is chairman of the EBM*Practice*Net board of editors. IK is the salaried Chief Editor of EBM Guidelines and EBMeDS decision support service. PVR is the chairman of the Primary Care Guidelines Working Group.

## Author contributions

ND, SVDV, MG and BF formalized and implemented the adaptation process. PVR supervised the process. ND drafted the manuscript. All authors commented on subsequent drafts and approved the final version.

## Supporting information


**Appendix S1** List of high‐priority guidelines.

**Appendix S2** Cholelithiasis.

**Appendix S3** Impetigo and other pyoderma.

**Appendix S4** Example Matrix.
