# Supplementary material for: Adapting a large database of point of care summarized guidelines: a process description
Source: J Eval Clin Pract. 2015 Aug 7;23(1):21–8. doi: 10.1111/jep.12426 (PMC5347856; doi:10.1111/jep.12426)
Supplement: Supplementary file 1 — Appendix S1 List of high‐priority guidelines. [file JEP-23-21-s001.docx]

List of high priority guidelines

| 1 | Dermatomycoses | 35 | Otitis externa |
| --- | --- | --- | --- |
| 2 | Hand and finger injuries | 36 | Cirrhosis of the liver |
| 3 | Gout | 37 | Clostridium difficile diarrhoea |
| 4 | Herpes zoster | 38 | Mycoplasma pneumoniae infections |
| 5 | Disorders of the rotator cuff of the shoulder | 39 | Lyme borreliosis |
| 6 | Low back pain | 40 | Hypothyroidism |
| 7 | Vertigo | 41 | Erysipelas |
| 8 | Deep vein thrombosis** | 42 | Diarrhoeal diseases caused by microbes |
| 9 | Heel pain | 43 | Hyponatraemia |
| 10 | Chronic obstructive pulmonary disease (COPD) | 44 | Disease-specific symptoms and signs in patients with inflammatory joint diseases |
| 11 | Wrist injuries | 45 | Examining a patient with a thyroid complaint |
| 12 | Acute coronary syndrome and myocardial infarction | 46 | Fractures of the foot |
| 13 | Cholelithiasis | 47 | Hypokalaemia |
| 14 | Acute abdomen in the adult | 48 | Mononucleosis |
| 15 | Chickenpox | 49 | Hypercalcaemia and hyperparathyroidism |
| 16 | Viral infections of the oral mucosa | 50 | Assessing a patient with an abnormal liver function test result |
| 17 | Conjunctivitis | 51 | Hernias in adults |
| 18 | Peptic ulcer disease, Helicobacter pylori infection and chronic gastritis | 52 | Hyperkalaemia |
| 19 | Osteoporosis | 53 | Hordeolum and chalazion |
| 20 | Nerve entrapment and compression disorders | 54 | Painful knee |
| 21 | Diverticulitis and diverticulosis | 55 | Skin diseases and the mouth |
| 22 | Hyperthyroidism | 56 | Insect bites and stings |
| 23 | Impetigo and other pyoderma | 57 | Achilles tendinopathy and tendon rupture |
| 24 | Dyspepsia | 58 | Addison's disease and other conditions inducing hypocortisolism |
| 25 | Anxiety disorder | 59 | Ankle fractures |
| 26 | Osteoarthritis | 60 | Ankylosing spondylitis and axial spondyloarthritis |
| 27 | Viral hepatitis | 61 | Balanitis, balanoposthitis and paraphimosis in the adult |
| 28 | Vaccinations* | 62 | Bite wounds |
| 29 | Knee injuries | 63 | Carpal tunnel syndrome (CTS) |
| 30 | Treatment of dyslipidaemias | 64 | Clinical assessment of memory impairment |
| 31 | Haemorrhoids | 65 | Coeliac disease |
| 32 | Haematuria | 66 | Coronary heart disease |
| 33 | Chronic inflammatory bowel disease | 67 | Enterovirus infections |
| 34 | Postmenopausal hormone replacement therapy | 68 | Erythema infectiosum |
| 69 | Temporal (giant cell) arteritis and polymyalgia rheumatica | 83 | Examination of the shoulder joint |
| 70 | Pulmonary embolism | 84 | Fungal infections of the mouth |
| 71 | Hives (urticaria) | 85 | Genital herpes |
| 72 | Benign paroxysmal positional vertigo (BPPV) | 86 | Hypocalcaemia, hypoparathyroidism and vitamin D deficiency |
| 73 | Urinary tract infection in a child | 87 | Leg oedema |
| 74 | Naevi and skin cancer (basal cell and epidermoid carcinoma) | 88 | Parkinson's disease |
| 75 | Benign prostatic hyperplasia | 89 | Paronychia and ingrown toenail |
| 76 | Psoriasis | 90 | Prolonged diarrhoea in the adult |
| 77 | Red, wet or sore eye | 91 | Prostate cancer |
| 78 | Rheumatoid arthritis | 92 | Rosacea |
| 79 | Seborrhoeic dermatitis | 93 | Skin abscess and folliculitis |
| 80 | Syncope: causes and investigations | 94 | Testis pain |
| 81 | Thrombocytopenia | 95 | Ulcerative colitis |
| 82 | Sports injuries and their prevention | 96 | Superficial venous thrombosis |

*item was omitted during the adaptation process due to issues of applicability

**item was processed twice
