# Supplementary material for: Adapting a large database of point of care summarized guidelines: a process description
Source: J Eval Clin Pract. 2015 Aug 7;23(1):21–8. doi: 10.1111/jep.12426 (PMC5347856; doi:10.1111/jep.12426)
Supplement: Supplementary file 2 — Appendix S2 Cholelithiasis. [file JEP-23-21-s002.docx]

This is the English version of the EBMG guideline Cholelithiasis which can be accessed by the EBM*Practice*Net.be website. All guidelines are available in Dutch and French. On the website they are presented in a slightly different layout including color codes for the references, grades of recommendations and hyperlinks to references and videos. The editor’s note is summarized in the text, and a hyperlink brings up a pop-up box with more information. In this example, the layout has been simplified and the editor’s note is shown as a text box.

**Cholelithiasis**

**Essentials**

- Identify patients whose pain is caused by gallstones and offer appropriate surgery.
- Complications are rare in asymptomatic gallstones, and surgery is not usually recommended.
- Acute cholecystitis and other complications of cholelithiasis (obstructive jaundice, suppurative cholangitis, empyema or gangrene of the gall bladder, enterobiliary fistula, gallstone ileus) should be treated as soon as possible after the onset of the symptoms **(GRADE A)**.
- Patients with cholelithiasis often have other illnesses (e.g. peptic ulcer, gastro-oesophageal reflux disease, lactose intolerance, coeliac disease, functional dyspepsia, irritable bowel syndrome, pancreatitis or even cancer). Any symptoms suggestive of the above illnesses usually warrant endoscopic, laboratory or imaging studies before surgery.

**Risk factors**

- Age
- Female sex
- Hereditary disposition
- Obesity
- Past deliveries
- Diabetes
- Hypothyroidism (especially common bile duct stones)
- Diseases of the ileum
- Total parenteral nutrition

**Clinical manifestation**

- Two-thirds of patients with gallstones are asymptomatic.
  - The pain often radiates into the shoulders or back. An attack is often accompanied by nausea and vomiting **(Johnson 2001)**.
    - Biliary pain lasting more than 12 hours with accompanying fever or jaundice is indicative of acute cholecystitis or cholangitis.

**Diagnosis**

- Ultrasonography is the investigation of choice for the diagnosis of both uncomplicated **(link to 2 videos)** and complicated cases. It detects stones in the gall bladder with over 90% sensitivity, but its sensitivity in detecting common bile duct stones is only 25%.
- Special investigations
  - ERCP (endoscopic retrograde cholangiopancreatography) may be used both for the diagnosis and extraction of common bile duct stones.
  - Increased serum concentrations of ALT, alkaline phosphatase and bilirubin, associated with an attack of pain, are indicative of common bile duct stones. However, about 40–60% of ERCP investigations, carried out on elevated liver function tests alone, turn out to be normal.
  - For the diagnosis of cholecystitis, and for the assessment of its severity, serum CRP and liver function tests (ALT, alkaline phosphatase, bilirubin) should be determined, in addition to clinical examination. Serum amylase concentration and an ultrasonography of the upper abdomen are used to exclude pancreatitis.

**Complications**

- Acute cholecystitis: biliary pain lasting more than 12 hours, fever and increased CRP
- Acute cholangitis: high fever, pain and jaundice
- Acute pancreatitis: severe pain, increased serum and urine amylase (or urine trypsinogen-2), increased liver function tests, history
- Jaundice
- Carcinoma of the gall bladder
- Gallstone ileus (a large gallstone passes into the duodenum through a cholecystoduodenal fistula and obstructs the bowel). The clinical picture is typical of intestinal obstruction. Plain abdominal x-ray may show air in the bile ducts.

**Indications and urgency of treatment**

- *Asymptomatic gallstones* need not be treated (with the exception of a totally calcified "porcelain" gall bladder which is associated with a markedly increased risk of cancer, immunosuppressive medication)**(Gibney 1990, Attili 1995)**.
- Patients with repeated episodes of *biliary pain* should be operated on within a few months, those with severe symptoms even more urgently. Pain-triggering foods should be avoided while waiting for the operation. NSAIDs or spasmolytics are given to alleviate the colicky pain.
- *Acute cholecystitis* should be treated surgically within 2–7 days from the onset of the symptoms**(GRADE B)**. Elderly patients and those in poor general health should also be referred for surgical evaluation. Intravenous fluids and analgesics are given for initial treatment. An antibiotic such as cefuroxime 1.5 g t.d.s., should be administered (the causative agent is usually E. coli).
- Patients with *acute biliary pancreatitis* must be immediately referred to hospital. Intravenous fluids and analgesics are given for initial treatment. MRCP (magnetic resonance cholangiopancreatography) or ERCP is carried out to verify the presence of common bile duct stones. If an impacted stone or cholangitis is detected during the urgent (within 48 hours) ERCP, a sphincterotomy and removal of the stone is carried out. Cholecystectomy is carried out within a month to prevent the recurrence of pancreatitis.
- A jaundiced patient must be referred to hospital for investigations and treatment within the next 24 hours.
- Carcinoma of the gall bladder is often an incidental finding during cholecystectomy. It is also occasionally diagnosed in patients with jaundice or other severe biliary symptoms. Individual decisions need to be made regarding further investigations and surgery.
- ***Editor’s note:*** *the recommendation for performing a cholecystectomy with 2 to 7 days after the onset of the symptoms seems arbitrary. More…*

*The recommendation for performing a cholecystectomy within 2 to 7 days after onset of the symptoms is based on several resources, including a Cochrane review, Clinical Evidence and a recent meta-analysis. Only Clinical Evidence mentions the period of 2 to 7 days. Both the meta-analysis and the Cochrane review state that early laparoscopic cholecystectomy lessens the hospitalization but lengthens the operation-time, without effect on complications or conversion to an open cholecystectomy. These studies do not mention how soon the cholecystectomy should be performed.*

**Current treatment trends and choice of methods**

- The complications of cholelithiasis should be treated with surgery within a few days of the onset of symptoms.
- Even very old patients and patients in poor general health can be treated with both conventional operative measures and with less invasive newer methods (radiological and endoscopic methods).
- Laparoscopic cholecystectomy is used increasingly for the removal of the gall bladder and common bile duct stones **(GRADE C)**. The benefits of this approach include shorter hospitalisation time and sick leave. A laparoscopic cholecystectomy must sometimes be converted to open surgery half way **(GRADE C)** through the procedure.
- Common bile duct stones can be removed with ERCP. In some cases it is possible to leave the gall bladder in situ in the elderly and in patients in poor general health. However, up to half of the patients will continue to experience attacks of pain, which will eventually lead to cholecystectomy in over a third of the patients **(Boerma 2002)**.
- Residual or recurrent stones after cholecystectomy can often be removed with ERCP.
- Asymptomatic gallstones are not removed surgically, as the operative risk (although minimal) exceeds the expected benefit.
- Gallstone dissolution and other experimental methods have not become routine practice as yet **(GRADE A)**.

**Abdominal pain after cholecystectomy**

- Abdominal pain after cholecystectomy may be caused by residual or recurring stones in the biliary tract, biliary strictures or spasms. Increased concentrations of serum ALT or alkaline phosphatase may suggest these conditions.
- The symptoms may have other than biliary aetiology, e.g. diseases of the stomach or colon (see the aforementioned list). Specialist investigations (endoscopy, imaging, laboratory investigations) should be carried out if necessary or the patient may need specialist consultation (always if laboratory tests are abnormal).

**Related resources**

- Cochrane reviews
- Other Internet resources
- Literature

Johnson CD. ABC of the upper gastrointestinal tract. Upper abdominal pain: Gall bladder. BMJ 2001 Nov 17;323(7322):1170-3.

Boerma D, Rauws EA, Keulemans YC, et al. Wait-and-see policy or laparoscopic cholecystectomy after endoscopic sphincterotomy for bile-duct stones: a randomised trial. Lancet 2002 Sep 7;360(9335):761-5.

Gibney EJ. Asymptomatic gallstones. Br J Surg 1990 Apr;77(4):368-72.

Attili AF, De Santis A, Capri R, Repice AM, Maselli S. The natural history of gallstones: the GREPCO experience. The GREPCO Group. Hepatology 1995 Mar;21(3):655-60.
