# Supplementary material for: Adapting a large database of point of care summarized guidelines: a process description
Source: J Eval Clin Pract. 2015 Aug 7;23(1):21–8. doi: 10.1111/jep.12426 (PMC5347856; doi:10.1111/jep.12426)
Supplement: Supplementary file 3 — Appendix S3 Impetigo and other pyoderma. [file JEP-23-21-s003.docx]

This is the English version of the EBMG guideline Impetigo and other pyoderma which can be accessed by the EBM*Practice*Net.be website. All guidelines are available in Dutch and French. On the website they are presented in a slightly different layout including color codes for the references, grades of recommendations and hyperlinks to references and videos. The editor’s note is summarized in the text, and a hyperlink brings up a pop-up box with more information. In this example, the layout has been simplified and the editor’s note is shown as a text box.

**Impetigo and other pyoderma**

**Aims**

- Impetigo should always be treated because it spreads easily in the family, day-care centre and school.
- Bacterial cultures and antibiograms should be used to determine antibiotic susceptibilities.
- Remember the possibility of post-infectious glomerulonephritis in streptococcal impetigo.

**Clinical features**

- Children are most commonly affected.
- Streptococcal infection typically makes crusts or small ulcerations; staphylococcal infection tends to make blisters **(links to 2 pictures)**.
- "Pemphigus neonatorum" **(link to picture)** in infants is actually impetigo. The infection is caused by S. aureus phage type II.
- The crusts usually appear in the surroundings of the nostrils, on the chin, and generally on the face **(links to 2 pictures)**.
- Thick crusts are characteristic.

**Differential diagnosis**

- Primary herpes simplex infection may resemble impetigo.
- Ringworm (Tinea corporis)
- If impetigo tends to recur in the scalp and neck consider the possibility of head lice.

**Causative agents**

- Staphylococcus aureus
- Group A beta-haemolytic streptococci **(link to picture)**
- Eczema may predispose the skin to impetigo.
- The infection usually spreads by autoinoculation.
- Recurrences are caused by bacteria remaining in the nostrils.

**Treatment**

- Treatment is started on the basis of the clinical presentation.
- If the disease is confined to a small area the treatment consists of soaking the crusts so that they are detached, and applying an antibiotic ointment (sodium fusidate **(GRADE A)** or a combination of neomycin and bacitracin e.g.).
- If the disease is more widespread (> 6 cm^2^) use a systemic antibiotic (first generation cephalosporin, e.g. cephalexin or cefadroxil 50 mg/kg daily for 7–10 days **(GRADE B)** or amoxicillin-clavulanic acid. Patients with cephalosporin allergy can be treated with clindamycin.
- Macrolides are no longer recommended.
- If the patient has eczema a topical preparation containing a corticosteroid and an antimicrobial agent should be used together with systemic antibiotics until the skin is intact. Do not forget further treatment of the eczema.
- The most common reasons for poor response to treatment:
  - The diagnosis is incorrect. The patient has scabies, lice, or ringworm.
  - The crusts have not been soaked and removed. The bacteria can survive under crusts.
  - The underlying eczema has not been treated.
  - The nostrils serve as reservoir for bacteria (apply neomycin-bacitracin or fusidic acid ointment into the nostrils. Mupirocin should not be used for this disease as its use should be limited to the eradication of methicillin-resistant Staphylococcus aureus)**(GRADE A)**.
- ***Editor’s note:*** *1. In case of limited lesions is a local treatment sufficient. 2. Oral antibiotics are recommended for more extensive lesions, insufficient effect from local treatment, adenopathy or systemic symptoms.*

Illustration (for 1):

First choice: fusidic acid 2%, 3 to 4 applications daily for 7 days (GRADE 1A)

Alternative: mupirocin 2%, 3 applications daily for 7 days (GRADE 2A). Muciprocin should be reserved for MRSA eradication.

Illustration (for 2):

First choice: flucloxacillin

-child: 25-50 mg/kg daily in 3 to 4 doses for 7 days

-adult: 1-2 g daily in 3 to 4 doses for 7 days

Alternative:

Clarithromycin

-child: 15 mg/kg daily is 2 doses for 7 days

-adult: 500 to 1000 mg daily in 2 doses for 7 days

Azithromycin

-child: 10 mg/kg daily in 1 dose for 3 days, or first day 10 mg/kg in 1 dose, then 5 mg/kg in 1 dose for 4 days

-adult: 500 mg daily in 1 dose for 3 days, or first day 500 mg in 1 dose, then 250 mg daily in 1 dose for 4 days

Roxithromycin: adult: 300 mg daily in 2 doses for 7 days

References: Chevalier P, Leconte S, De Sutter A. Belgian guide for antimicrobial treatment in the ambulatory practice, BAPCOC, 2008.

**Related resources**

- Other Internet resources
- Literature
